# The nutritional environment and food access of female athletes at HBCUs: a qualitative analysis

**DOI:** 10.3389/fnut.2025.1675069

**Published:** 2026-01-09

**Authors:** Nancy A. Uriegas, Zachary K. Winkelmann, Dawn M. Emerson, Toni M. Torres-McGehee

**Affiliations:** 1 Department of Health and Human Performance, Texas State University, San Marcos, TX, United States; 2 Department of Exercise Science, Univeristy of South Carolina, Columbia, SC, United States; 3 School of Kinesiology, Physical Education, and Athletic Training, Univeristy of Maine, Orono, ME, United States

**Keywords:** food security, nutritional habits, diversity, campus food environment, college health

## Abstract

**Purpose:**

Female student-athletes attending Historically Black Colleges and Universities (HBCUs) may encounter distinct challenges related to food access, particularly when their campuses are situated in areas with limited food availability. This study assessed food security (FS) and explored the lived experiences of HBCU female student-athletes regarding their food environments and access to nutrition.

**Methods:**

This qualitative study used a semi-structured interview protocol derived from quantitative data. Participants were 10 HBCU female athletes (age = 19 ± 1 years) living in on-campus dormitories; recruited after completing a quantitative study examining energy needs. The three-part semi-structured interview protocol included questions about personal experiences surrounding food access and the U.S. Department of Agriculture Six-Item Short Form of the Food Security Survey Module. Interviews were recorded and transcribed verbatim. Three coders analyzed data using the consensual qualitative research tradition. Trustworthiness was established using member checking, multi-analyst triangulation, and external auditing.

**Results:**

FS status was evenly distributed among participants (five food secure, five food insecure). Four domains emerged: (1) on-campus living, (2) personal factors, (3) convenience, and (4) nutritional awareness. Participants described navigating limited cooking resources, inconsistent cafeteria offerings, and time constraints that influenced their food choices. While food sources were available both on and off campus, many participants expressed that these options did not consistently align with their preferences or nutritional needs. Reflections on dietary habits revealed varying levels of nutritional awareness and a desire for more tailored guidance.

**Conclusion:**

The findings suggest that while food options exist within HBCU campuses and surrounding communities, they may not fully support the unique needs of female student-athletes. Institutional stakeholders, including administrators, athletic trainers, and coaches, may consider collaborative efforts to enhance food access and nutritional support. This could include increasing awareness of community resources (e.g., SNAP, food banks), revisiting dormitory policies related to cooking, and expanding access to nutrition education. These insights underscore the importance of contextually responsive strategies to promote student-athlete well-being.

## 1 Introduction

For student-athletes, adequate dietary intake, including a balance of macronutrients and micronutrients, is imperative for performance and wellness. Researchers have identified female athletes as an at-risk group for the female athlete triad (Triad) and relative energy deficiency in sport [REDs; (1, 2)]. Female athletes' predisposition to these conditions centers around low energy availability (LEA), typically due to high training volumes and under fueling. Other contributors to inadequate dietary intake include barriers to adequate nutrition, lack of nutrition resources, lack of motivation to fuel appropriately, and body image dissatisfaction (3). Despite growing recognition of REDs and LEA, little is known about how food insecurity (FI) and constrained food access uniquely manifest for female athletes attending Historically Black Colleges and Universities (HBCUs), where institutional and geographic factors may further complicate fueling and recovery.

Social needs relative to fueling focus on food access and FI. FI is defined as “limited or uncertain availability of nutritionally adequate and safe foods or limited or uncertain ability to acquire acceptable foods in socially acceptable ways” (4). In the U.S., 17 million households are food insecure, with 6.8 million facing very low food security [FS; (5)]. Rates of FI among postsecondary students have recently become a concern; across colleges, 61% of students had low FS, and 57% had very low FS (6). College students are particularly vulnerable to FI due to financial constraints, limited transportation, and inconsistent access to food. Student-athletes represent an especially at-risk subset given their elevated nutritional demands, rigid schedules, and restricted food choices. Yet, the prevalence and lived experience of FI among student-athletes, particularly those attending HBCUs, remain underexplored.

Existing studies estimate FI prevalence ranging from 10% to 60% across the National Collegiate Athletic Association (NCAA) divisions, with notable disparities by sex and division level (7–10). For instance, 14.7% of NCAA Division III athletes reported FI (7), while only 9.9% of male athletes at a Division I institution did (9), compared to 32% of female athletes at the same level, with no significant racial differences (8). These findings highlight the disproportionate burden of FI across athletic contexts and underscore the need for more nuanced, intersectional research.

FI is a multifaceted and complex issue influenced by individual, social, and environmental factors and is linked to inadequate dietary intake, which can compromise health, sleep, mental well-being, and athletic performance. While prior research has documented FI's impact on student-athletes, most studies rely on quantitative data and do not explore how student-athletes of color experience food access within the unique cultural and institutional context of HBCUs. Although the relationship between race and FI has been well-established (11), few studies have centered HBCUs or examined food access through a multidimensional lens.

To address this gap, our study examined FS among female student-athletes at HBCUs and explore their lived experiences surrounding the food environment and ability to access food following the examination of their energy needs where data indicated low dietary intake. Our qualitative exploration was guided by the five dimensions of food access, availability, accessibility, affordability, accommodation, and acceptability, originally developed in health services research and later adapted to food environments (12, 13). These dimensions informed our interview protocol and analysis, allowing us to examine food access in a culturally responsive and context-specific way. Prior work by Andress and Fitch (14) similarly highlighted how structural, economic, and cultural factors shape food access among low-income individuals, themes that may resonate with the experiences of student-athletes navigating FI at HBCUs. In the context of collegiate athletics, food access is not only a matter of availability or affordability; it directly influences an athlete's ability to fuel adequately for performance and recovery. For female student-athletes, especially those at HBCUs, the intersection of food access and fueling is shaped by institutional resources, cultural relevance, and economic constraints. By examining food access through this lens, our study captures how structural and personal barriers impact both FA and the capacity to meet energy demands.

## 2 Materials and methods

### 2.1 Research design

Our study followed a sequential mixed-methods design; this portion focused on the qualitative data. We followed the consensual qualitative research (CQR) tradition with semi-structured interviews to explore HBCU female student-athletes' experiences with their food environment and access based on the five dimensions of food access: availability, accessibility, affordability, accommodation, and acceptability. The Standards for Reporting Qualitative Research were used to guide the methodological approach to the reporting process (15). The Institutional Review Board at University of South Carolina approved this study (Pro00133920).

### 2.2 Participants

Following the completion of the quantitative study (16), 13 of 29 participants expressed interest in participating in the interviews, and data saturation was achieved after 10 interviews. Table 1 presents demographic information, including pseudonyms and classification of low food access areas. The classification of low food access areas surrounding HBCU campuses was based on the USDA Economic Research Service Food Access Research Atlas (17), which identifies census tracts characterized by limited access to food stores and low household income.

**Table 1:** Participant demographics.

**Pseudonym**	**Age**	**Ethnicity**	**Academic status**	**Sport**	**Scholarship status**	**Living accommodations**	^*^Low income/low food access	**Food security**
Sophia	18	Hispanic	Freshman/1st yr	Soccer	Partial athletic	On-campus suites	Yes/Yes	High
Andell	19	Black	Sophomore/2nd yr	Soccer	Partial athletic	On-campus suites	Yes/Yes	Low
Nikki	19	Multiethnic	Sophomore/2nd yr	Soccer	Partial athletic	On-campus suites	Yes/Yes	Low
Constance	20	Black	Junior/3rd yr	Soccer	Partial athletic	On-campus suites	Yes/Yes	Very low
Tiffany	20	Black	Junior/3rd yr	Volleyball	Partial athletic	On-campus suites	Yes/No	Very low
Niecy	21	Black	Senior/4th yr	Volleyball	Full athletic	On-campus suites	Yes/No	Marginal
Camille	18	Black	Freshman/1st yr	Volleyball	Partial athletic	On-campus dormitory	Yes/No	High
Veronika	18	Black	Freshman/1st yr	Volleyball	Full athletic	On-campus dormitory	Yes/No	Marginal
Chandra	19	Black	Freshman/1st yr	Basketball	Partial athletic	On-campus dormitory	Yes/Yes	High
Kimberly Ann	21	Black	Junior/3rd yr	Basketball	Partial athletic	On-campus suites	Yes/Yes	Low

^*^Areas of low income/low food access as defined by the USDA Economic Research Service Food Access Research Atlas using 2019 data.

### 2.3 Interview protocol

An interview protocol was developed connecting the two phases of this project. Interview questions, specifically, were guided by data from the participant's dietary logs. The interview protocol included an introduction, additional demographic information, and a three-part semi-structured set of questions about personal experiences surrounding food access. Part one of the interview focused on the five dimensions of food access previously defined by Andress and Fitch (14). The second portion of the interview included the United States Department of Agriculture (USDA) Six-Item Short Form of the Food Security Survey Module and a reflection of their FS status (18). The USDA six-item short form module has demonstrated internal consistency and validity (Cronbach's alpha > 0.80) in comparison to the full 18-itme scale (18). FS was categorized according to the USDA scoring protocol, raw scores of 0 = high FS; 1 = marginal FS; 2–4 = low FS, and 5–6 = very low FS. The last portion focused on the individual participant's dietary logs. Follow-up questions were utilized to ensure participants provided the most complete and accurate information. The interview protocol was developed based on existing literature and refined by the study authors, who specialize in nutrition, food security, and qualitative methods, and reviewed for content relevance and clarity. A pilot interview was conducted with a non-participant student who shared similar demographics and had completed the dietary intake logs discussed in the interview. Feedback from the pilot informed a round of revisions to improve question phrasing and flow. The final version of the protocol is presented in Table 2.

**Table 2:** Interview protocol.

Demographic questions	1 What financial assistance is currently available to you as a student-athlete? 2 What are your housing accommodations during the school year? 3 Do you currently have a meal plan through your institution
Five A's to food access	1 Please describe the availability of food options on or near your campus. 2 Can describe how you access the food options on or near your campus. a Do you have any trouble getting to places to buy food? b Do you have any barriers to consuming the food that is available to you? 3 Next, I want to talk to you about the accommodations of the food options. Do you feel the sources of food respond to your needs? c Describe how the dining hall and other on-campus options address your nutritional needs as a student-athlete. d Describe how off-campus options in the community address your nutritional needs as a student-athlete. e Can you discuss the hours of the dining hall? 4 Tell me about the actual food you consume on campus. Is it acceptable to your liking? f Do you feel the food options align with your cultural background? 5 Lastly, I would like you to tell me more about purchasing the food that is available to you. (Affordability) g Is it affordable based on your income?
Food security module and reflection	I am going to read you several statements that people have made about their food situation. For these statements, please tell me whether the statement was often true, sometimes true, or never true for *YOU* in the last 12 months—that is, since last (name of current month). 1 The first statement is, “The food that I bought just didn't last, and I didn't have money to get more.” Was that often, sometimes, or never true for you in the last 12 months? 2 “I couldn't afford to eat balanced meals.” Was that often, sometimes, or never true for you in the last 12 months? 3 In the last 12 months, since last (name of current month), did you ever cut the size of your meals or skip meals because there was not enough money for food? 4 *[IF YES ABOVE, ASK]* How often did this happen—almost every month, some months but not every month, or in only 1 or 2 months? 5 In the last 12 months, did you ever eat less than you felt you should because there was not enough money for food? 6 In the last 12 months, were you every hungry but didn't eat because there wasn't enough money for food? 7 Based on those questions, you would be considered to have [*food security statu*s], which is defined as: [*definition*]. What do you feel is the main reasons for this to be true? a How do you plan to navigate this or improve it? What is your ideal situation?
Dietary log reflection	The last portion of the interview is for you to consider the 7-day study you did with me. 1 Can you reflect on the process of logging your food in the diary. *Individualized per person, based on review of dietary log*. 2 I noticed…. [*insert info*]—could you help me to understand your thought process with these decisions or choices. 3 Do you feel your dietary intake meets the demands of your physical activity? 4 Since then, have you noticed any changes or modifications to your diet or nutritional intake? 5 Please describe, if any, the benefits you experienced from participating in the study?

### 2.4 Procedures

Participants who expressed interest in participating in the interviews received an electronic invitation, which included the purpose, expected duration of interview, and the risks/benefits associated with the study. Participants were then directed to an online scheduling platform that automatically emailed a link to a web-conferencing (Zoom, San Jose, CA) meeting and a calendar invite. A follow-up email including the participant's dietary log, was sent. Interviews were approximately 30 min and were audio-only recorded for transcription purposes. The audio files were downloaded and transcribed verbatim using otter.ai technology embedded within Zoom. After transcription, the primary investigator reviewed the transcript and audio files to deidentify information and check accuracy. Lastly, each participant received their transcript for member checking, participants ensured accuracy and were allowed to clarify and/or update responses. One participant provided written changes to their responses, while others expressed the information was correct as is.

### 2.5 Data analysis

Data were analyzed using the three central steps of the CQR tradition: domains, core ideas, and cross-analysis (19). A team of three researchers (NAU, ZKW, TMTM) with varying experience coded the data. To begin, the coding team reviewed three transcripts separately and identified core ideas. The coding team then met to discuss core ideas and establish a preliminary codebook, including the emergent domains and categories. Next, the preliminary codebook was applied to two previously reviewed transcripts and two new transcripts to establish validity. The coding team met again to discuss converging and diverging opinions; no changes were made to the codebook. Upon finalization of the codebook, each member independently coded 3–4 transcripts. Transcripts were exchanged across coders for an internal review. After the internal review, all transcripts were reviewed and voted on coding discrepancies using a two-thirds agreement. Finally, an external auditor with extensive qualitative experience reviewed the work utilizing the codebook in three selected transcripts.

A frequency classification count was assigned for each emerging category: *general* = 9–10, *typical* = 6–8, *variant* = 3–5, *rare* = 1–2 cases for this sample (19). Trustworthiness and credibility were ensured using member checking, multiple-analyst triangulation, and external auditing. The research team also performed a reflexivity check to control our bias during the coding process. The coding team has an extensive research background in feeding and eating disorders, lived in college dormitories, and participated in college athletics as an athlete or athletic trainer; however, none attended HBCUs. We acknowledge that this positionality may have shaped our interpretation of participants' experiences, particularly regarding institutional culture, racialized context, and food access at HBCUs. To mitigate outsider bias, we engaged in consensus coding, maintained reflexive memos throughout analysis, and held regular team discussions to critically examine assumptions and ensure cultural responsiveness. We also prioritized participant voice by grounding interpretations in direct quotes and contextualizing findings within existing literature on FI and HBCU environments.

## 3 Results

FS status varied across participants, resulting in an even distribution between FS (*n* = 5, 50%) and FI (*n* = 5, 50%). Additionally, four domains emerged: (1) on-campus living, (2) personal, (3) convenience, and (4) nutritional awareness. Categories further specified each domain to illustrate the experiences of the participants. Frequency counts are presented in Table 3.

**Table 3:** Frequency counts.

**Domain and category**	**Frequency *n* = 10**	**CQR assigned value**
**On-campus living**		
Housing resources	10	General
Cafeteria options	10	General
**Personal**		
Budgets	9	General
Teammate support	7	Typical
Preferences	10	General
**Convenience**		
Off-campus resources	10	General
Timing conflicts	10	General
Quick options	10	General
**Nutritional awareness**		
Prior knowledge	10	General
Fueling habits	10	General

### 3.1 On-campus living

Within the on-campus living domain, student-athletes described their *housing resources* in terms of the set-up of their dormitory and access to kitchen/cooking supplies and the *cafeteria options* available through the on-campus dining facilities.

#### 3.1.1 Housing resources

All female student-athletes lived in on-campus housing; however, the set-ups varied by institution and academic level, with participants having at least one roommate or suitemate. Overall, participants did not have access to a full-size kitchen to make their meals but described having access to refrigerators and microwaves. Those who lived in suite-style dormitories discussed having a common living area where they would keep small kitchen appliances to attempt to make some meals but were unsure what the dormitory policies were for having these appliances. Sophia, a sophomore soccer athlete, shared, “*We have a full fridge, but we do not have a stove. We have an air fryer in there, a microwave, and one of those rice cookers.”*

However, this was not the case for some of the younger student-athletes, who discussed that as 1st-year students living on-campus, it was extremely challenging to keep food in their rooms, and they look forward to the change when they transition out of their 1st year. Camille mentioned:

“*We have a microwave, but it is not in our rooms. It is on the 12*^*th*^ *floor of the building and everyone shares it. We cannot have any electric stuff to make any type of food, but this is only for the freshmen…next year should be different.”*

#### 3.1.2 Cafeteria options

Student-athletes spoke extensively about the dining options available on their campuses, which included institutional cafeterias and limited franchise options. Participants were enrolled at three different NCAA Division I HBCUs, each with a central dining hall. While all campuses offered multiple food stations, hours of operation varied and often included closures of 1–3.5 h between mealtimes, which shaped athletes' ability to access food consistently throughout the day.

Descriptions of cafeteria layout and offerings reflected the availability dimension of food access. Andell described the structure of her campus dining hall:

“*When you first step into the dining hall, you get greeted with a calendar and have two sides [to choose from]. The right side is the grill area, a fried [food] area, and they have pizza. Then, to your left, they have subs, salads, fruits, and the international station… The fried station they usually do burgers, sandwiches, and fries. It usually stays pretty greasy… The international, which you are not going to know what you are going to get, sometimes it is open, but for the majority of the year it is not, so I just do not even bother going over there.”*

While multiple stations were technically available, athletes noted that some were inconsistently open or offered limited variety, reducing the practical availability of diverse, nutrient-dense options. Chandra shared that menu rotation was predictable but narrow, “*Wednesdays are strictly fried chicken and then Fridays, they typically try to have fish.”* This repetition of fried or processed foods across campuses suggests that while food was present, it may not have been acceptable in terms of nutritional quality or cultural relevance. Athletes often gravitated toward a preferred station that met their personal standards for taste and fueling. Tiffany described her go-to option:

“*I am a huge fan of the stir fry line which offers your choice of protein, whether it is chicken or steak or some type of beef, and then they have different vegetables, onions, carrots, spinach, and a lot of other options, and rice. They cook it in front of you, so I would say, that is one of my favorite options. If I can I usually try to stop there very often, and then I sometimes get the pizza as well.”*

### 3.2 Personal

The personal domain included factors influencing student-athletes' food decisions. The participants discussed *budgets, teammates' support*, and their *preferences* when selecting foods.

#### 3.2.1 Budgets

Student athletes shared mixed experiences regarding the affordability of food options both on and off campus. While all participants reported having access to a meal plan for on-campus dining, many noted that these plans did not consistently meet their needs or preferences. Some meal plans covered only a limited number of meals per day and may have restricted dining hours, making them difficult to use regularly. As a result, participants often sought alternative food sources, such as grocery shopping, dining out, or preparing their own meals, which introduced additional financial strain, as Constance reflected,

“*If I want to cook and eat healthy regularly, I prep my own food, then that probably requires I go to the grocery store, maybe 3 or 4 times out of the month. Whatever the case may be, to keep getting the stuff I need, and at that point it becomes not affordable. So, yes, I would say, grocery shopping is not affordable because you can get some stuff, but you always have to re-up and get more, and that is the expensive part.”*

Additionally, participants shared that financial situations are different amongst their teammates. In our sample, some participants disclosed having part-time jobs, while others shared their parents/guardians supported them financially throughout the year and, therefore, they may be restricted by the parents'/guardians' financial situation. Kimberly Ann talked about this by sharing:

“*Sometimes I will have to wait, my mom gets paid on certain days, so I wait till then. If I had to pay for it by myself, I would be starving, but my mama pays. Sometimes, she will say I have to wait till she gets paid to squeeze it in, and there are times my dad will send me like $15. However, there are times where I will go [out to eat] and do not get anything because I do not have the money for it.”*

#### 3.2.2 Teammate support

Female student-athletes talked about the camaraderie among their teammates. Their roommates typically played the same sport, meaning they shared mealtimes and played a role in their decisions. As for Chandra, this was an essential factor when deciding to go to breakfast:

“*I am not very independent so, sometimes when I do get up in the morning to go breakfast, I do not want to go by myself. It is actually one of the only reasons I would not go to breakfast. My roommate is not an early bird, so the decision to skip meals was because I did not want to go by myself.”*

Moreover, having access to a vehicle on campus appeared to be restricted based on academic status, requiring many to catch rides from others or walk to food and grocery options. Sophia discussed her experience as a 1st-year student trying to go off-campus for groceries:

“*Freshmen are not allowed to have our cars on campus, so within our dorm there are at least 2 girls in each room that have a car, and we are all friends. So, if they are going to the grocery store, they will ask if we want to come with them, so I have never had a problem where I am running out of food or other stuff. We go to the store pretty often. I would say twice a week at least.”*

#### 3.2.3 Preferences

Participants were asked whether the food available on campus met their cultural and nutritional needs. Their responses reflected a mix of personal preferences, flavor expectations, and perceptions of food quality. While some student-athletes found certain meals enjoyable, others described the food as bland, unfamiliar, or visually unappealing. Kimberly Ann shared, “*Sometimes the taste is a little too bland, but it usually is in the cafeteria. I like it when they have chicken, rice, and broccoli. The broccoli bussin'!”*

This quote illustrates how athletes formed preferences based on taste and familiarity, though the colloquial enthusiasm also underscores the rarity of meals that felt satisfying or culturally resonant. Across institutions, participants described relying on visual cues and past experiences to guide their food choices. For example, one athlete recounted being served a version of shepherd's pie that was unfamiliar in appearance and taste, prompting her to seek food elsewhere.

These reflections highlight the acceptability dimension of food access, as student-athletes often evaluated meals not only for nutritional adequacy but also for cultural relevance, flavor, and presentation. When meals did not meet these standards, athletes frequently opted for off-campus alternatives, suggesting that institutional dining may fall short in meeting the nuanced preferences of this population.

### 3.3 Convenience

The next domain to emerge following the interviews was convenience. Student athletes described the off-campus resources available in their community, the *timing conflicts* they faced, and some of the *quick options* they relied on to fuel throughout the day.

#### 3.3.1 Off-campus resources

Options within the community primarily included fast food and some grocery stores. Student athletes discussed primarily relying on fast food options when they did not want to eat at the on-campus dining facilities. Camille described some of the options in her community and how she accesses them:

“*There is a Food Lion right by the school, but it is a good walk. There is a Kentucky Fried Chicken, a Subway, and a barbecue place very close to the school, too. Then there is a Chick-fil-A, a Waffle House, and a Cookout, but they are a good walk away. Those are the places that I have eaten from while I have been here. I normally walk because I do not have a vehicle right now, to me it is not a far walk, but people who are not used to walking will probably say it is pretty far.”*

Student athletes who had vehicles felt they could explore further but had to think about balancing the options and finances. Niecy shared her experience:

“*I eat a lot of fried food and fast food. In the area that we are in, you will have to leave and drive 10-15 minutes away to get more of a healthier meal, which is obviously going to be at a higher price. So, you have to pick and choose, ‘am I going to eat Mexican food [at a restaurant] today, or am I going to go to Cookout today?' You have to have a balance.”*

#### 3.3.2 Timing conflicts

One of the primary challenges to fueling discussed by student-athletes was time. Given the hours of operation of their on-campus dining facilities, student-athletes shared their classes or practice times often interfered with getting to the dining facilities. Niecy provided some insight to her personal situation:

“*I usually do not eat breakfast, then most of my classes are around one and sometimes I have meetings around lunch. So, I might just have to pick something up really fast or just have something in my room if I want to eat during that time. Practice is normally a little later in the day, it starts around 6pm or so and we finish at like 8pm. Everything around here closes at 9 or 10 pm, so if you have to hurry up and shower and get out to pick something up once you get out of practice.”*

Following the FS questionnaire, Constance shared some thoughts on what might make her personal situation better,

“*Something that I think would help, would be increasing the amount of time for lunch and dinner. I am not really a breakfast person, but if the amount of time we had at our cafeteria was increased for lunch and dinner, I feel like I would be able to make it more often. Most times when I go to the cafeteria, I can find something to eat, so I feel like that would improve everything for me.”*

#### 3.3.3 Quick options

Snacks and microwaveable meals were among some of the quick options student-athletes described purchasing at the grocery store to have on the go. Options ranged from granola bars, breakfast tarts, and microwaveable noodles to frozen meals. Female student-athletes mentioned carrying snacks in their bag on busy days or because they rather have smaller options, as shared by Andell:

“*I try to pick up things for early morning practice because I know I am going to need something to eat. I get small things like fruits or pre-packaged fruits and protein bars. I always make sure to get snacks for in between classes.”*

Chandra, a first-year collegiate basketball player, shared similar thoughts:

“*I try to keep snacks in my room just in case I cannot get to the cafeteria. I could just stop by my dorm to grab a quick snack or a little something to eat to hold me over until the next time I can get to the cafeteria. I keep granola bars, chips, juice, and water.”*

### 3.4 Nutritional awareness

The final domain to emerge from the interviews was nutritional awareness. When asked specific questions following the quantitative study (16), athletes reflected on their *fueling habits* and detailed having some nutritional *knowledge sources*, which appeared to have been informal conversations.

#### 3.4.1 Fueling habits

After completing the quantitative study (16), specific questions were formulated for each interviewee based on their detailed dietary reports. We specifically asked the participants if they thought they were fueling properly and had observed any benefits after participating in the study. Nikki shared, “*I think it made me aware of skipping meals. It made me see how all over the place my eating is. Sometimes a little more healthy, sometimes unhealthy.”* Fueling for their sports appears to be challenging for some participants, and they recognize that they are not eating well. At times participants shared the reason behind not fueling is simply that they do not feel hungry or may choose to sleep over fueling. Veronika recognized some of her habits and how she could do better: “*I just feel like I should have more meals instead of snacks, maybe have more balanced meals and three meals a day. Maybe I should try to get up and go to breakfast, too.”*

#### 3.4.2 Knowledge resources

Lastly, student-athletes identified knowledge resources they had been exposed to, specifically nutrition. Through our interviews, we captured that the three institutions did not have a sports dietitian or a designated staff member whose primary responsibility was nutrition. Student athletes mentioned coaches, high school teachers, athletic trainers, and strength and conditioning specialists as individuals who provide nutritional knowledge. Many times, it was general information and not specific to the individual or their sport. Kimberly Ann discusses a prior experience:

“*Last year at my junior college, we had a little segment, but I feel like it has never been broken down. I just know to have the energy to perform, I need to be eating, but there has never been formal training. No, never.”*

Furthermore, Tiffany recalled a similar experience:

“*Within our weight training class, our trainer went over how to eat certain foods and what foods to eat to fuel. I am not sure of specifics, but it was mainly on our fruit and vegetable intake, as well as matching the protein with the amount of calories we are burning.”*

## 4 Discussion

Access to healthy food among college students enrolled in HBCUs has not been widely documented and less is known about the experience of food access among HBCU student-athletes. To our knowledge, few studies have qualitatively explored how FI and constrained food access intersect with athletic identity, institutional context, and fueling demands, particularly among student-athletes of color. Existing research has examined FI among general college populations, including students at minority-serving institutions (20, 21), but studies focused specifically on HBCU athletes remain limited. The findings of our study suggest that while food sources are present on campus and in surrounding communities, they may not consistently meet athletes' personal standards, cultural preferences, or time constraints, making it difficult to fuel adequately for sport and recovery.

### 4.1 On-campus living

Female student-athletes shared their experiences living on campus and how it may affect the options available to fuel properly. Student athletes typically relied on the on-campus dining facilities to fuel. On-campus dining facilities offer an array of options to suit the needs of students. However, student-athletes engage in additional training that may require more specialized diets. Previously, students have been dissatisfied with the menu variety, food quality, and healthy options available at university dining facilities (22). Student athletes in our study echoed these sentiments by sharing their dislikes with the dietary options available, citing the flavor and appearance of food as unpleasant.

Furthermore, as described by the participants, dormitories are typically not conducive to establishing good dietary patterns. While the dormitories where the student-athletes lived differed from traditional dormitories, none of the participants had access to a full kitchen to prepare their meals, a feature many of them expressed a strong desire for. Several of the participants reported using small kitchen appliances such as hot pans, rice cookers, and pressure cookers to cook for themselves. However, using these appliances often violates dormitory guidelines, putting students at risk for disciplinary action. Recognizing these challenges, some colleges have begun revising their housing policies to allow certain small kitchen appliances, acknowledging the value of fostering independent living skills (23).

We recommend that colleges and universities consider updating their housing policies to allow for safe use of small kitchen appliances without fear of repercussions, as it may support student well-being and autonomy. At the same time, we understand that fire safety is a concern of the administration (24). To balance these priorities, institutions should explore ways to upgrade dormitories with full-size kitchens or design communal kitchen spaces to ensure students have the means to adequately meet their nutritional needs. These findings highlight the importance of institutional support in addressing the unique dietary needs of student-athletes and suggest that improvements in housing and dining infrastructure may contribute to better health outcomes, academic success, and athletic performance.

### 4.2 Personal

The 2020 #RealCollege Survey documents that two out of every three HBCU students experienced basic needs insecurity, with 46% facing FI (25). Demographic, behavioral, and mental factors have significant associations with FI among HBCU students compared to students attending a private university or community college. Using a validated FS screening tool, we identified 50% of the student-athletes in this study had FI. As previously mentioned, the participants discussed financial constraints, affecting the dimensions of affordability, accommodation, and acceptability, similar to the experiences shared by low-income residents (14).

Although all participants received athletic scholarships (Table 1) and had access to institutional meal plans, these supports did not fully eliminate food access challenges. Scholarships often cover tuition and fees but may not provide sufficient stipends for groceries or off-campus meals, especially when athletes seek options that align with their nutritional needs. As student-athletes progress in college to upper division (e.g. junior, senior), it is likely that many may move off-campus for housing. While meal plan access may be available, some student-athletes may experience food security issues preparing their own meals at their off campus housing. Likewise, meal plans were described as limited in scope; some only covered a set number of meals per day or operated during restricted hours, which often conflicted with athletes' training and academic schedules. These limitations highlight the need for institutions to go beyond financial aid and meal plans by improving access to supplemental resources and tailoring dining services to meet the unique needs of student-athletes.

Students, including student-athletes, have previously reported they may utilize on-campus resources better than federal programs such as the Supplemental Nutritional Assistance Program [SNAP; (26)]. Campuses across the U.S. have increasingly established community food assistance centers, often termed food pantries or food banks, to support students experiencing food insecurity, and to our knowledge, such resources are available at the institutions attended by our participants. However, this topic was not raised during the interviews, so it remains unclear whether the student-athletes are aware of or utilize these options. While we cannot draw conclusions about their awareness of the use of food assistance programs, this absence suggests a potential gap in visibility or accessibility that warrants further investigation.

Additionally, the application process for SNAP benefits may pose a barrier for students. Colleges and universities might consider offering targeted support services to help students navigate this process. Student-athletes also shared that they often relied on teammates for transportation to grocery stores and for sharing meals. These interactions may play a role in shaping eating behaviors and attitudes, and prior research suggests that peer support can be protective against disordered eating behaviors among student-athletes (27). Future studies could explore how informal support networks interact with institutional resources to influence food access and dietary patterns.

Some participants expressed strong aversions to the campus cafeteria food, citing issues such as limited variety, unappetizing options, and concerns about food safety. While these preferences and concerns are common among college students, it is important to acknowledge that, in rare cases, persistent and extreme food avoidance may align with characteristics of Avoidant/Restrictive Food Intake Disorder (ARFID), a clinical condition recognized in the DSM-5 (28). However, the data from this study do not provide sufficient evidence to suggest the presence of disordered eating patterns. Rather, these findings likely reflect typical food preferences and environmental dissatisfaction, which may still impact students' overall well-being and dietary habits.

### 4.3 Convenience

Birkenhead and Slatern (29) stated that athletes' food choices are potentially influenced by pressure to perform, body image concerns, the impact of exercise on appetite, and exposure to unique food environments. HBCU student-athletes, in particular, are often exposed to food environments shaped by limited access to nutritious options. Prior research has shown that many HBCUs are located in low food access areas, geographic regions with restricted availability of full-service grocery stores (30, 31). For example, only 24% of food stores within a 15-mile driving radius of HBCUs in North Carolina are considered favorable (e.g., supermarkets, produce vendors), and just four such stores were identified within a 15-min walking distance (31). These environmental constraints often leave students reliant on limited on-campus dining or nearby fast-food options, which are typically less nutritious.

Compared to public non-HBCUs, a higher proportion of HBCUs (46.9%) are situated in low-access food areas, further compounding the challenge of maintaining a balanced diet. In our study, female student-athletes frequently cited fast food as their most accessible off-campus option, underscoring the influence of the surrounding food environment on their dietary habits. While these limitations were not always explicitly framed as a concern, participants' narratives revealed how constrained food availability shaped their daily fueling decisions.

In addition to environmental barriers, student-athletes face time and financial constraints that further complicate their ability to access nutritious meals. Demands such as class schedules, athletic training, employment, and social obligations often limit the time available for meal preparation or travel to grocery stores (32, 33). Participants in this study described relying on quick, convenient options like snacks or frozen meals, despite recognizing that these choices often fell short of meeting their nutritional needs. This aligns with findings from Anziano and Zigmont (34), who reported student-athlete support for extended dining hall hours to better accommodate their schedules.

Given these challenges, institutional and municipal interventions could play a critical role in improving food access for HBCU student-athletes. Potential strategies include establishing partnerships between universities and local grocers or farmers' markets to bring fresh produce and healthy options to campus. Implementing shuttle services to nearby supermarkets or community food hubs could also alleviate transportation barriers. Additionally, expanding campus dining hours and offering mobile meal options tailored to athletes' schedules may help address time-related constraints. These efforts could contribute to more equitable food environments and support the health, performance, and academic success of student-athletes at HBCUs.

### 4.4 Nutritional awareness

Within the domain of nutritional awareness, female student-athletes acknowledged challenges in adequately fueling for the demands of their sport and reflected on their sources of nutritional information. Participation in the quantitative component of the study (16) served as an experiential learning opportunity, prompting many to reflect on their eating habits and energy needs. To better understand these reflections, the conscious-competence model (35) can serve as a useful interpretive lens. Rather than a strict, linear progression, this model offers a flexible framework for understanding how individuals may move between different levels of awareness and skill related to nutrition.

Some participants appeared to describe experiences aligned with *unconscious incompetence*, in which they were unaware of the extent to which their dietary habits were misaligned with their physical demands (35). For others, the process of tracking dietary intake and energy expenditure during the study brought about a shift toward *conscious incompetence*, a growing awareness of nutritional gaps and the realization that their current habits were insufficient. Several student-athletes discussed intentions to improve their eating behaviors, such as increasing fluid intake, eating three meals a day, or incorporating more fruits and vegetables. These efforts reflect aspects of *conscious competence*, where individuals begin to apply new knowledge, though doing so may still require deliberate effort (35).

Student-athletes may fluctuate between levels of awareness and competence depending on context, support systems, and environmental constraints. Achieving *unconscious competence*, where healthy fueling becomes second nature, may require sustained access to nutrition education, institutional support, and structural changes to the food environment. Recognizing this fluidity allows for a more nuanced understanding of how student-athletes develop nutritional awareness and the types of interventions that may support them.

While many of the participants shared receiving information previously, it appears this was done informally or were general dietary guidelines rather than specific to their physical activity. Sports nutrition knowledge has been widely examined across NCAA athletes, with findings suggesting knowledge levels are poor and do not meet current recommendations (33, 36–40). The studies previously conducted include an array of athletic divisions. However, it is unclear whether HBCU student-athletes participated. To our knowledge, only one study has been previously presented on HBCU student-athlete's knowledge, with mean knowledge scores yielding 38.82 ± 4.1 out of 110 points, meaning nutrition knowledge is poor in this population (41). Female student-athletes discussed athletic trainers, coaches, teachers, and strength and conditioning specialists as sources of knowledge. Athletic trainers (71.4%) and strength and conditioning specialists (83.1%) have been found to have adequate nutrition knowledge; however, 64.1% of coaches had inadequate knowledge, suggesting they may not be the best source of nutrition information for student-athletes (40). Data suggest the incorporation of sports-registered dietitians into athletic teams provides benefits to student-athletes (33, 42). Specifically among female athletes, Valliant et al. (33) reported individualized education focused on increasing the knowledge of types and amounts of food based on the individual and their needs made significant improvements in knowledge and overall dietary intake including increases in carbohydrates and protein. Allowing student-athletes access to sports dietitians would be beneficial across HBCUs; if not available, athletic trainers and strength and conditioning specialists should receive additional training to assist athletes with more individualized dietary information. Additionally, HBCU athletic departments could explore the use of telehealth and mobile applications maintained by registered sports dietitians, which would allow their student-athletes to interact with trained providers (43).

### 4.5 Limitations and future research

While this study provides valuable insight into the food environment and nutritional experiences of female student-athletes at HBCUs, several limitations should be acknowledged. First, the sample was limited to participants from institutions within a single state, which may restrict the generalizability of findings to other geographic regions or institutional contexts. Additionally, the study relied on self-reported dietary logs, which are subject to recall bias and may not accurately capture actual intake. The small sample size, while appropriate for qualitative inquiry, also limits the extent to which findings can be generalized. Furthermore, participants may have been influenced by social desirability bias, particularly when discussing health behaviors such as eating habits and nutrition knowledge. Finally, while efforts were made to ensure the trustworthiness of the interview guide through expert review and iterative refinement, we acknowledge that the study did not employ a formal Content Validity Index (CVI) process. The CVI process is increasingly recognized in qualitative research as a valuable tool for enhancing the rigor and validity of data collection instruments. Future research should consider incorporating the CVI procedures to further strengthen the methodological integrity of qualitative interview protocols.

Despite these limitations, the study offers important exploratory insights and highlights areas for future research. Expanding this work to include male student-athletes, athletes from a broader range of sports, and participants from diverse HBCU settings would enhance understanding of the unique challenges and opportunities related to nutrition and food access in these communities. Future studies might also consider incorporating objective dietary assessments and longitudinal designs to better capture changes in behavior and awareness over time.

## 5 Conclusion

The present study explored the food environments and dietary practices of HBCU female student-athletes at HBCUs. Throughout our interviews, we learned female student-athletes have various food options available at their institutions and within their communities; however, factors such as sports timing, class schedules, and financial constraints posed barriers to adequately fueling. FS status varied among participants, indicating that not all student-athletes experience these challenges equally. To better support their well-being and performance, student-athletes would benefit from individualized nutritional education programs and greater access to community resources and public benefits such as SNAP benefits and food banks. Facilitating awareness of and access to these resources is crucial. College and universities can play a pivotal role by streamlining these processes and fostering partnerships with local organizations to bring services directly on campus. Such collaborations have the potential to reduce FI, promote healthier eating habits, and enhance the academic and athletic success of HBCU student-athletes.

## Data Availability

The raw data supporting the conclusions of this article will be made available by the authors, without undue reservation.
